# Multiple thyroid nodules in the lung: metastasis or ectopia?

**DOI:** 10.1186/s13000-015-0299-8

**Published:** 2015-06-06

**Authors:** Henghui Cheng, Lili Yang, Jing Xiong, Jian Peng, Qiurong Ruan

**Affiliations:** Institute of Pathology, Tongji Hospital, Tongji Medical College, Huazhong University of Science and Technology, 1095 Jiefang Dadao, Wuhan, 430030 People’s Republic of China; Department of Radiology, Tongji Hospital, Tongji Medical College, Huazhong University of Science and Technology, Wuhan, People’s Republic of China

**Keywords:** Thyroid Cancer-clinical, Thyroid Nodules, Pathology-Thyroid

## Abstract

**Background:**

Intrapulmonary thyroid tissue with no malignant history of the thyroid gland is extremely rare. Usually, it is interpreted as ectopic thyroid tissue. Here we describe a case of bilateral pulmonary thyroid nodules with a history of multinodular thyroid goiter.

**History:**

A 37-year-old female had recurrent multinodular thyroid goiter and showed bilateral pulmonary nodules on CT scan. Video-assisted thoracic surgery (VATS) was performed for the largest nodule biopsy. Pathological and molecular examinations were done after biopsy, and both were shown the characters of benign thyroid tissues. To eliminate the possibility of thyroid carcinoma metastases, total thyroidectomy with modified radical neck dissection was performed, and there were no malignant pathological findings. After surgery, this patient accepted adjuvant radiometabolic treatment for ablation of the remaining intrapulmonary nodules. Her thyroglobulin level decreased to an undetectable level, and she has currently survived for 24 months after surgery.

**Clinical significance:**

In this case, pulmonary ectopic thyroid and metastasizing thyroid carcinoma should both be considered, but the metastatic pattern and benign pathological characters were inconsistent with any of the corresponding diagnosis. Ultimately, this patient accepted postoperative treatment of thyroid carcinoma metastasis.

**Conclusions:**

This is a rare thyroid disease with malignant behavior but no pathological evidence. Careful diagnosis and postoprative follow-up should be carried out whenever such nodules are encountered in clinical practice.

**Virtual slides:**

The virtual slide(s) for this article can be found here: http://www.diagnosticpathology.diagnomx.eu/vs/1255194331453728.

## Background

Thyroid benign nodules arising from follicular cells, including multinodular goiter, are generally thought not to metastasize. Therefore, once there are benign lesions in regional or distant organs, ectopic thyroid tissue should initially be considered. Usually, aberrant thyroid tissue can be found in the midline from the tongue to the diaphragm, along the thyroglossal tract [1]. While thyroid ectopia in the lung is uncommon, the possibility of thyroid carcinoma should also be considered. Since this is a lesion distinct from ectopic thyroid, treatment and prognosis would be significantly different. One should be cautious in making the diagnosis, especially when the metastases or/and the primary lesion have no obvious malignant pathological morphology. Here we report a rare case of multiple pulmonary thyroid nodules, concomitant recurrent multinodular goiter of the thyroid gland. The interpretation of such a finding may be of critical importance in the diagnosis and the ensuing therapeutic intervention.

## Methods

The study was approved by the Ethics Committee of Tongji Hospital, Tongji Medical College, Huazhong University of Science and Technology, Wuhan, China. Written informed consent was obtained from the patient.

Serum levels of thyrotropin (TSH), thyroglobulin (TG), free triiodothyronine (FT3), and free thyroxine (FT4) were measured by chemiluminescent immunoassays (Abbott system).

Surgical specimens were fixed in 10 % neutrally buffered formalin and embedded in paraffin. Sections were deparaffinized by xylene and then rehydrated. The histological morphology was examined after staining with hematoxylin and eosin. Immunostaining was performed through a two-step Envision method with microwave-citrate antigen retrieval. Diaminobenzidine was used as the chromogen. Nuclei were stained with Mayer’s hematoxylin. Appropriate positive and negative controls were included.

Total RNA and genomic DNA were extracted from paraffin-embedded tissue samples which were macrodissected to remove stromal contamination and to ensure tumor cellularity of ≥80 %. Polymerase chain reaction (PCR) and reverse transcription (RT-PCR) were both done, according to the manufacturer’s recommendations. BRAF Pyro Kit (QIAGEN) was used for BRAF mutation in codon 600. Quantitative evaluation of RET/PTC1 and RET/PTC3 rearrangements by real-time PCR were performed by an ABI PRISM 7700 Sequence Detection System as described in a previous report [2]. In the study, PTC tissues with known RET/PTC1 and RET/PTC3 rearrangements served as a reference standard.

## Case presentation

A 37-year-woman was admitted to our hospital for the third time due to a recurrent left neck mass. She underwent two resections of the left thyroid mass at the ages of 29 and 33, respectively, and both of the left thyroid masses were removed and underwent careful pathological examinations. There was multinodular colloid goiter with macrocalcification, and no malignant cells or follicular adenoma were found, which was in accordance with ultrasonography before resections. This time, four years after the second surgery, the multiple nodules in the remnant thyroid enlarged again. Fine needle aspiration was carried out and showed no malignancy. Neck ultrasonography was performed and a multiple nodular thyroid tissue with macrocalcification was noted in the left thyroid lobe. Her thyroid function tests indicated that she was euthyroid. The serum TSH was 1.38uTU/mL (0.30–4.94), the FT3 was 3.26 pg/mL (1.71–3.71) and the FT4 was 1.23 ng/dL (0.70–1.48). But the TG level was elevated to 326.3 ng/ml (3.50–77.00).

**Fig. 1:**
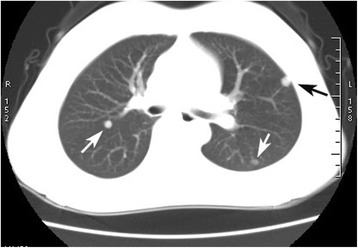
On lung window image of the chest computed tomography (CT) scan, three 3–5 mm-sized, well-defined, round nodule are seen in the bilateral pulmonary lobes, and one nodule is revealed beneath the pleura (black arrow)

**Fig. 2:**
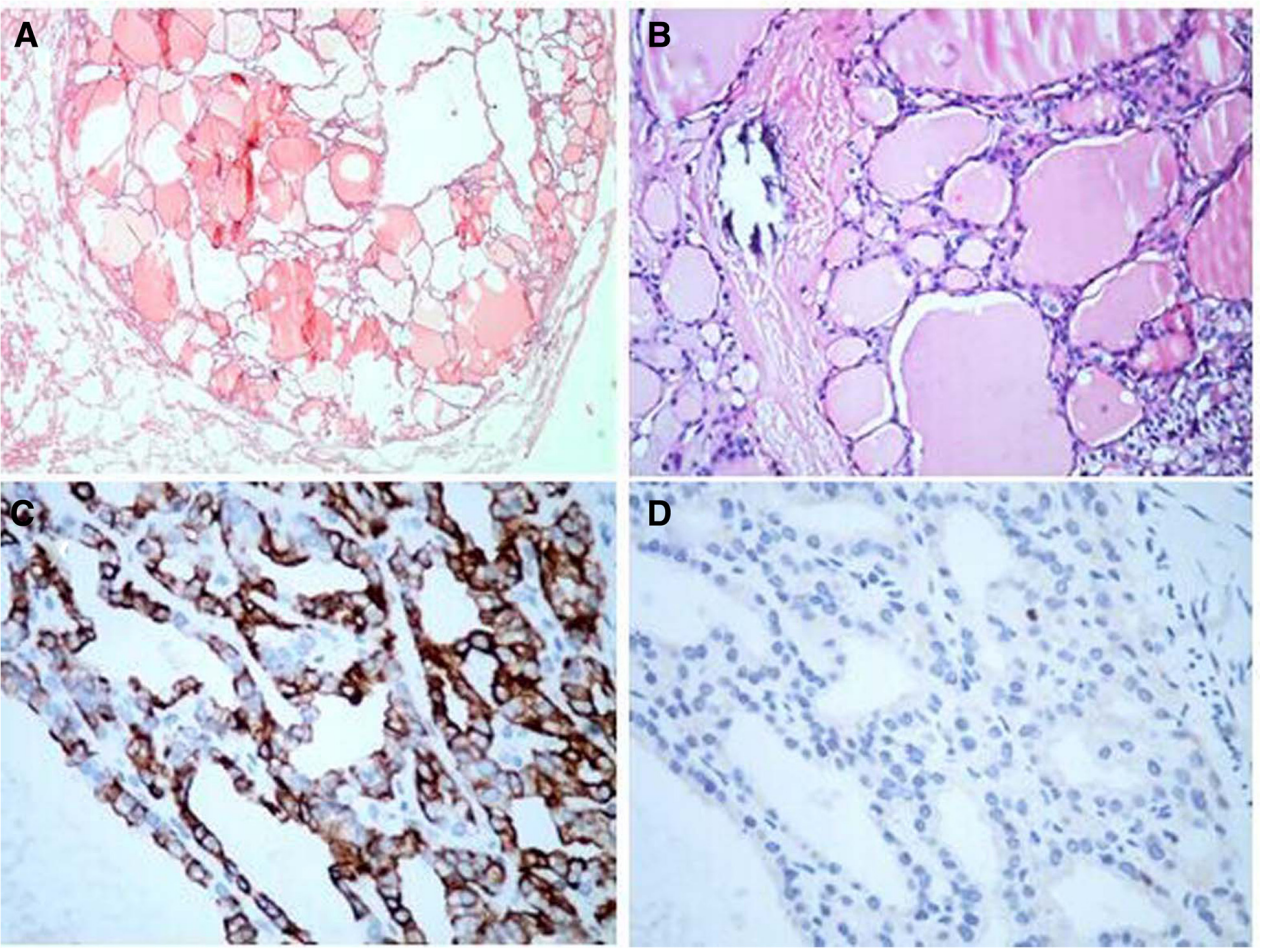
**a** A well-circumscribed thyroid tissue in the pulmonary parenchyma (hematoxylin and eosin, 20 × magnification). **b** The nodule contains various-sized follicles with no cellular atypia (hematoxylin and eosin, 200 × magnification). **c** The immunohistochemistry staining of follicular epithelial cells showed strong positive for thyroglobulin (TG, 400 × magnification). **d** Negative immunohistochemistry staining for Galectin-3 (400 × magnification)

Computed tomography (CT) scans suggested that there were several well-defined, round, tiny nodules, ranging from 3 to 5 mm in diameters, in the bilateral lung lobes (Fig. 1). In order to verify the diagnosis, video-assisted thoracic surgery (VATS) was used to biopsy the largest nodule in the lung. The nodule was well demarcated from the surrounding lung tissue, without an obviously fibrous capsule. Histologically, this focus was composed solely of various-sized colloid-filled thyroid follicles (Fig. 2). These follicles lined by a single layer of flattened to cuboidal epithelial cells without atypia. Immunohistochemical staining for both thyroglobulin (TG) and thyroid transcription factor-1 (TTF-1) showed strong reactions within the epithelial cells. CD56, a thyroid follicular epithelial cell marker, was also positive. CK19, HBME-1 and Galectin-3, which are usually positive in papillary carcinoma, were all negative (Fig. 2).

**Fig. 3:**
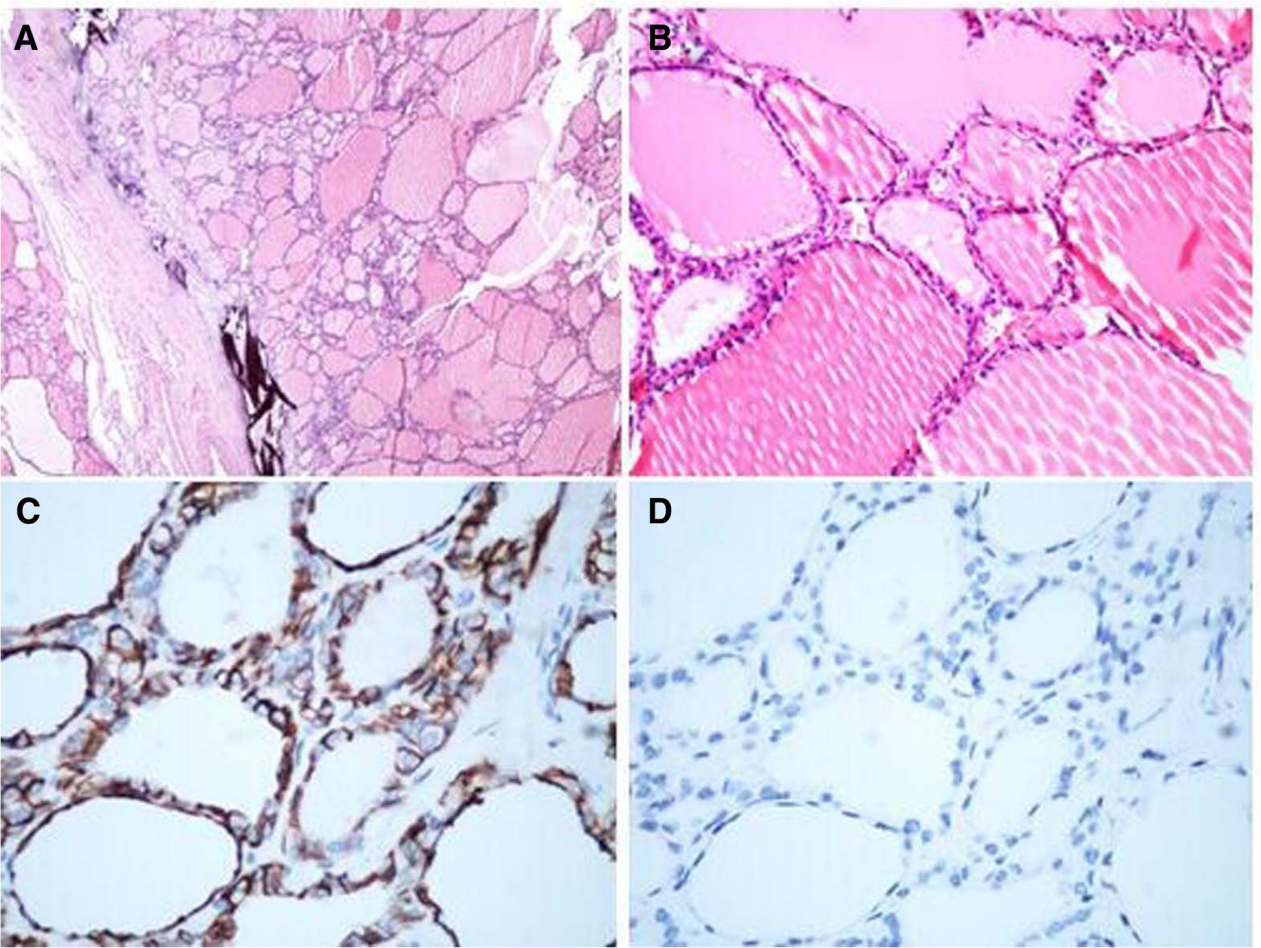
**a** The thyroid multinodular goiter contains normal thyroid follicles with calcification but no psammoma bodies (hematoxylin and eosin, 40 × magnification). **b** The follicles epithelial cells showed no atypia (hematoxylin and eosin, 200 × magnification). **c** The CD56 immunohistochemistry staining reveals a positive reaction of follicular epithelial cells (400 × magnification). **d** Negative immunohistochemistry staining for Galectin-3 (400 × magnification)

Subsequently, the patient underwent a total thyroidectomy with modified radical neck dissection to remove the thyroid mass. On gross examination, only the superior lobe of the left thyroid remained, about 1.5 cm × 1.0 cm, soft and dark red, multinodular colloid, and the right thyroid tissue was homogenous, without any nodules. The whole left thyroid gland was cut and embedded for histological morphology examination. Microscopically, left thyroid nodules were composed with distinctive follicles filled with colloid, like the intropulmonary nodule (Fig. 3). No follicular adenoma or capsular/vascular invasion was found. The lining cells were flattened or cuboidal, and lacked nuclear features of papillary carcinoma. Fibrous tissue hyperplasia and calcification could be found in the left thyroid. There were no particular findings in the right thyroid. Immunohistochemical staining was also performed on the left thyroid and the results were exactly the same to those in the intropulmonary nodule (Fig. 3).

**Fig. 4:**
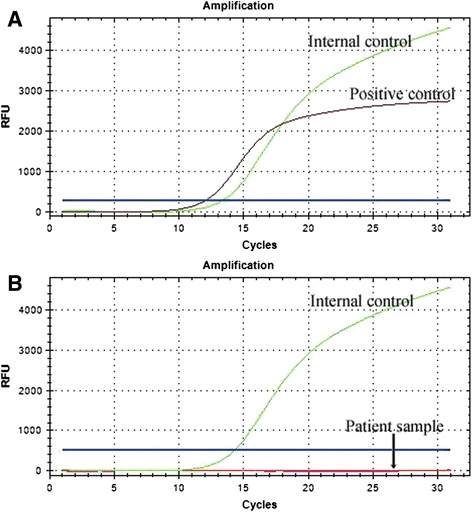
Real-time polymerase chain reaction of the BRAFV600E mutation analysis was performed. **a** The positive control clearly shows the BRAFV600E mutation. **b** The BRAF mutation is not detected in the patient pulmonary nodule

Molecular assays were carried out on both the lung nodule and the left thyroid to detect the possibility of malignancy. The BRAFV600E mutation, including the common mutational hotspots was not identified in the DNA extracted from these tissues compared with papillary carcinoma (Fig. 4). Expression of RET/PTC1 and RET/PTC3 genes were also assessed and neither RET/PTC1 nor RET/PTC3 was demonstrated in the present case.

Considering the recurrent history of thyroid mass and the bilateral distribution pattern of pulmonary nodules, the surgeon ultimately decided to carry out the treatment for metastasizing thyroid carcinoma. Subsequently, TSH suppression therapy and radioiodine therapy for ablation of the lung metastasis were done. The patient’s thyroid function was nearly within the normal range, except for a slightly high level of the serum thyrotropin (8.41uTU/ml). The sizes of those nodules remaining in the lung were reduced. She had an uneventful course of recovery without remarkable findings after a follow-up for 24 months.

## Discussion

Intrapulmonary benign thyroid tissue is very rare, with only seven cases reported so far [3-9]. Most of these cases were interpreted as ectopic thyroid, because they were found either during the work-up of a solitary pulmonary nodule [4,7] or at autopsy without evidence of a primary thyroid gland tumor [6,9]. And the nodules were always single [3-6,9]. The diagnosis of intropulmonary ectopic thyroid should be made cautiously, based on exclusion of any kind of tumor from thyroid gland. In this case, there was not only a definite long history of left thyroid mass, but also bilateral multiple pulmonary nodules on ultrasonography, which were highly suspected to be metastasizing. As a result, the possibility of ectopic thyroid of the lung seemed less likely than metastasis. Though rare, different ectopic foci of ectopic thyroid gland can be present simultaneously. In a recent review, Sood A et al. reported 27 cases of dual ectopic thyroid gland [10]. Only one case of simultaneously bilateral intropulmonary ectopic thyroid has been reported before [7].

To clarify the possibility of metastasizing thyroid carcinoma, all the tissues of the left thyroid gland and pulmonary thyroid nodule were cut out and embedded for careful pathological examination. No malignant features, such as papillary structure, follicular cell atypia, psammoma bodies, and so on, were found on the left thyroid samples or the biopsy lung nodule. Furthermore, immunohistochemistry, BRAFV600E mutation feature, and RET/PTC1 or RET/PTC3 gene expression were all consistent with the histomorphology. Other organs, such as the lymph nodes and bone, did not show any metastasis or other abnormality.

Nevertheless, the possibility that the multiple intrapulmonary thyroid nodules may represent metastasizing thyroid carcinoma could not be excluded. Occult thyroid carcinoma is sometimes undetectable on ultrasonography or on palpation, and is usually overlooked, especially combined with other nodular lesions, such as multinodular goiter [11]. Therefore, the possibility of occult thyroid carcinoma in the left thyroid tissue existed. But occult thyroid carcinoma preferentially metastasizes to the regional lymph nodes and rarely to distant organs [12]. So if this case was metastizing thyroid carcinoma, the question arose as to how the distant metastasis in the lung and the benign pathological characteristic of pulmonary metastatic lesions might be explained. In fact, according to the literature, there were several cases in which it was reported that occult thyroid carcinoma could metastasize to distant organs, including the lung [7]. Research was also conducted about the morphology of metastatic lesion of thyroid tumors. In continuously cultured thyroid tumor cells [13] and thyroid tumor cells injected in vivo to mimic metastasis [14], the cultured cells lose tumor cell characteristics in certain conditions, and the metastatic lesion in certain organs only showed benign morphology in animals. These results suggested that the malignant features of the metastatic lesion disappear in certain conditions. Ito Y et al. had been reported five cases of occult thyroid carcinoma with distant metastasis but no malignant pathological findings in both thyroid gland and metastatic lesions [8]. These patients all had good prospects after radiotherapy. Therefore, occult thyroid carcinoma with benign looking introplumonary metastatic nodules is extremely rare, but not impossible.

In this case, the possibility of pulmonary metastasis from struma ovarii was also significant, because malignant struma ovarii sometimes display the pulmonary metastasis in women [15]. However, malignant struma ovarii would show malignancy in the intrapulmonary nodule. Furthermore, the bilateral adnexal ultrasonography did not show ovarian enlargement. Therefore, malignant struma ovarii could be excluded in this case.

Although the patient had a recurrent history of multinodular goiter, high thyroglobulin level and bilateral pulmonary goiter nodules, she ultimately accepted the management of metastasizing thyroid carcinoma. We still, however, have doubts that this case was represented a thyroid carcinoma coexisting with lung metastasis or simultaneous bilateral intropulmonary ectopic thyroid tissues. Recently, the Foxe1 mutation is known to be associated with the molecular pathology of ectopic thyroid in mouse models; however, no known gene for human ectopic thyroid has been demonstrated [16]. There are still limitations in diagnosing thyroid carcinoma, especially follicular thyroid carcinoma or occult thyroid carcinoma.

## Conclusions

We think this is an exceedingly rare and interesting case, which may inspire debates about methods of precise diagnosis and management for cases that lacking malignant pathological evidence but having malignant clinical behavior. Since some metastatic lesions of thyroid carcinoma can hide malignant pathological characteristics, and occasionally ectopic thyroid nodules occur multifocally, these complex diseases may easily be misdiagnosed when encountered in the clinical setting. Since thyroid carcinoma and ectopic thyroid tissue have different treatment and prognoses, more focus should be given to recurrent multinodular goiter, and extended follow-up of this kind of cases should be further conducted.

## Consent

Written informed consent was obtained from the patient for the publication of this report and any accompanying images.
